# Exploring and Modifying BarrieRs to enhance ACcess to mental health support for Ethnic minority Children and Young People (CYP) in acute paediatric settings (EMBRACE) in England: a realist review protocol

**DOI:** 10.1136/bmjopen-2025-104145

**Published:** 2026-01-22

**Authors:** Morenike Da-Silva-Ellimah, Natalie Darko, Yasuhiro Kotera, Kapil Sayal, Joseph C Manning

**Affiliations:** 1 School of Healthcare, College of Life Sciences, University of Leicester, Leicester, UK; 2 Leicester NIHR Biomedical Research Centre, College of Life Sciences, University of Leicester, Leicester, UK; 3 School of Health Sciences, University of Nottingham, Nottingham, UK; 4 Centre for Infectious Disease Education and Research, Osaka University, Osaka, Japan; 5 School of Medicine, Mental Health and Clinical Neurosciences, Institute of Mental Health, University of Nottingham, Nottingham, UK; 6 Nottingham Children’s Hospital, Nottingham University Hospitals NHS Trust, Nottingham, UK

**Keywords:** Child & adolescent psychiatry, Paediatric A&E and ambulatory care, PAEDIATRICS, Health Services Accessibility

## Abstract

**Abstract:**

**Introduction:**

Globally, up to 15% of children and adolescents experience a mental health (MH) condition. In the UK, an increasing number of children and young people (CYP) are presenting to acute paediatric settings (paediatric emergency departments and paediatric medical wards) with MH issues. However, the literature suggests that the MH support available in acute paediatric settings is insufficient and unsafe in England. A key principle in NHS England’s plan for joint working to support CYP with MH needs in acute paediatric settings is for care to be personalised to the needs of CYP. However, there is a paucity of research that explores the needs of ethnic minority CYP with MH issues in acute paediatric settings, and recent research has highlighted racial disparities in the accessibility and outcomes of MH services for CYP. This is significant as MH issues in childhood are associated with lifelong inequalities in health, employment, education and mortality outcomes in later life. We aim to explore how, why and under what circumstances acute paediatric settings support (or do not support) ethnic minority CYP to access appropriate MH support, and to develop a refined programme theory for the important contextual factors and mechanisms that can influence whether acute paediatric settings support ethnic minority CYP in accessing appropriate MH support.

**Methods and analysis:**

This review will use the realist approach developed by Pawson and Tilly which involves six steps: (1) Clarifying the scope of the review, (2) Searching for evidence, (3) Selecting and appraising the primary studies, (4) Extracting and organising the data, (5) Analysing and synthesising the findings and (6) Disseminating the findings. We will search OVID Medline, PsycINFO, CINAHL and SCOPUS. Relevant data will also be sought through snowballing and backward citation searching on included studies, seeking document recommendations from relevant professionals, and grey literature searches on Grey Matters, Health Management Information Centre and Google Scholar. The search will cover documents published from database inception. Documents featuring Black and/or Mixed-Black CYP with MH issues in acute paediatric settings will be included. Documents that do not separately report the results of CYP (<18 years old) from Black ethnic groups, or are unavailable in English will be excluded. An advisory group of key stakeholders will be recruited and involved throughout all stages of the review to promote the design and outputs of the realist review reflecting the experiences of (a) Ethnic minority CYP with MH issues and (b) The professionals involved in their care and the acute paediatric setting. The output of this process will be a refined middle-range theory that will provide a detailed understanding and explanation of the key contextual factors and mechanisms involved in ethnic minority CYP accessing MH support.

**Ethics and dissemination:**

This realist review will only involve secondary data, so ethical approval will not be required. The developed programme theory will be disseminated through the advisory group, peer-reviewed publications, discussions with relevant stakeholders and presentations at relevant research conferences and community events. Additionally, the theory will inform a primary realist evaluation study where the theory will be tested and refined further.

**PROSPERO registration number:**

PROSPERO, CRD420251009912.

**STRENGTHS AND LIMITATIONS OF THIS STUDY**

- The realist review will explore the mental health (MH) support needs of ethnic minority children and young people (CYP), which is required to address ethnic inequalities in mental healthcare.
- The realist methodology will provide novel insight into what MH interventions in acute paediatric settings are likely to work for ethnic minority CYP, for whom and in what context.
- The involvement of an advisory group during the review will support the usefulness and relevance of the findings to clinical practice and key stakeholders.
- The explanations of the context-mechanism-outcome relationships of the MH interventions can be used to learn from previous challenges, inform policy and guide further exploration through realist evaluation studies.
- This is a novel research area, and the usefulness of the results will depend on the availability of relevant literature on the topic.

## Introduction

 Mental health (MH) conditions have a substantial burden on children and young people (CYP), and their families globally. It has been estimated that at least 8% of children and 15% of adolescents worldwide have a MH condition.^1 2^ MH refers to a state of mental well-being that allows individuals to cope with life stressors, fulfil their abilities, work well, learn well and contribute to their community.^3^ MH disorders reflect clinically significant disturbances in cognition, behaviour and/or emotional functioning.^4^ MH conditions encompass MH disorders, psychosocial disabilities and any other mental states that are linked to significant distress, impaired functioning or a risk of self-harm.^3^ Individuals can have problems or issues with their mental well-being that do not meet the criteria for a MH condition.^5^

The developing brains of CYP are particularly susceptible to positive and negative influences, making childhood and adolescence sensitive periods for MH.^6^ A third of MH conditions emerge by the age of 14, and half by the age of 18.^6^ Anxiety, depression and behavioural disorders are the leading causes of disability and illness in adolescents, and suicide is the third leading cause of death in 15–29 year-olds globally.^2^ This is significant because MH issues in childhood are associated with lifelong inequalities in health, employment, education and mortality outcomes in later life.^7^,^9^ The United Nations Children’s Fund and WHO’s service guidance for CYPs MH states that MH services should be inclusive to children of all backgrounds.^6^ The guidance recognises that no single model for CYP’s mental healthcare can be applied to all contexts, and it encourages each country to strengthen the design and delivery of their MH services for CYP.^6^

Focusing on England, an increasing number of CYP are experiencing MH issues, 20% of CYP have a probable MH disorder, and since 2017, the proportion of 8–16 year-olds with a probable MH disorder has increased by 8%, and the number of 17–19 year-olds by 13%.^10^ The common MH disorders in England include emotional disorders such as depression, anxiety, phobia, panic disorders and obsessive-compulsive disorder.^11^ Additionally, the prevalence of eating disorders among 11–16 year-olds was eight times higher in 2023 (2.6%) than in 2017.^10 12^ MH issues among CYP in England were considerably exacerbated by COVID-19, Child and Adolescent Mental Health Services (CAMHS) referrals increased by 81% between 2019 and 2021, and the number of CYP accessing MH services rose by 15.5% between December 2021 and December 2023.^7 13^ However, the demand for CAMHS services outweighs the supply, so CYP can experience delays in accessing these services.^14^

There are persistent and longstanding ethnic inequalities in access to MH support, with higher rates of MH issues but poorer rates of access and outcomes among ethnic minority CYP in England. There is no internationally standardised definition of the term ‘ethnic minority’, but it is defined by the United Nations as an ethnic or racial group in a country where they are not the dominant ethnic population.^15^ A secondary analysis of the MH of CYP in England survey revealed that CYP with MH concerns aged 5–16 years, from Black (11.7%), Asian (55.1%) and Mixed (46.0%) ethnic groups reported less MH-related contact with professional services than CYP from the White ethnic group (66.9%).^16^ Additionally, Black men are more likely to access MH services in acute crisis, and in 2023 Black people in England were 3.5 times more likely than their white counterparts to be detained under the Mental Health Act (MHA).^17 18^ Acute MH presentations require more intensive and usually more expensive treatment, representing a significant socioeconomic cost to individuals, society and the NHS.^17^ Ethnic minority CYP also have poorer access to MH support in the US. Elliott *et al* found Hispanic and Black children were less likely to receive MH treatment than their white counterparts, after adjusting for MH symptoms.^19^ Also, an international meta-analysis found Black and South Asian individuals aged 13 and over were more likely to be admitted involuntarily under the MHA than their white counterparts.^20^ These inequalities in access cannot be explained by cultural differences alone. They have been attributed to discrimination, racism, language barriers, socioeconomic disadvantage, a different understanding of MH problems, a lack of information about services, a lack of trust in healthcare professionals and cultural expectations about mental resilience.^21 22^ These inequalities need to be addressed as the Race Relations (Amendment) Act 2000 mandates healthcare institutions to ensure racial equality in their service provision.^23^ This issue has been recognised as a national health priority in NHS England’s Core20PLUS5 approach to reducing health inequalities for CYP, which lists improving access to MH services for CYP from ethnic minority groups as an area requiring accelerated improvement.^7^

Traditionally, the management of CYPs’ MH conditions has been attributed to CAMHS, and the management of their physical health conditions to Paediatrics services.^24^ However, an increasing number of CYP are accessing MH support through acute paediatric settings. This includes emergency departments and paediatric medical wards. In 2022, 12% of NHS paediatric admissions in England were for a MH concern, representing a 65% increase from 2012.^25^ Also, the national Far Away From Home study of adolescents admitted to out-of-area or adult MH units found that of the adolescents who had to wait for a bed, 40% waited in paediatric wards and 7.2% in the emergency department, often for a week or more.^26^ CYP can wait in acute paediatric settings for a MH assessment and/or treatment because the MHA regards the hospital as a ‘place of safety’.^27^ Additionally, CYP with MH issues may be in acute paediatric settings for medical stabilisation or a co-morbid physical health need, as having a physical illness increases the risk of mental illness by 82%.^28 29^

Despite the hospital being regarded as a place of safety, the literature suggests that the MH support available in acute paediatric settings is inadequate.^28^ The MH support available includes support from CAMHS Liaison teams, MH champions and signposting to community MH services.^30^ The Health Services Safety Investigations Body deemed paediatric wards unsafe for managing high-risk MH behaviours after finding CYP had little to no specialist MH care and therapeutic engagement, and that the measures put in place to promote physical safety were restrictive, which increased the risk of conflict situations.^30 31^ Limited staff training and staffing levels are also factors that contribute to inadequate MH support on paediatric wards.^31 32^ In a survey of CAMHS provision in emergency departments in the UK, some emergency departments reported wait times of 12–24 hours for a CAMHS assessment.^33^ In 2022, NHS England released a plan for joint working to support CYP with MH needs in acute paediatric settings, and a key principle in the plan is for care to be personalised to the needs of CYP.^28^ However, there is a paucity of research that explores the needs of ethnic minority CYP with MH issues in acute paediatric settings. This is significant because a cohort study of all admissions to medical wards in England for CYP aged 5–18 years old from 2012 to 2022 showed an 81% increased likelihood of a MH admission lasting longer than 1 week for CYP in the Black ethnic group compared with those in the White ethnic group.^25^ Research which explores the MH needs of ethnic minority CYP in acute paediatric settings is therefore vital to enable solutions to be developed which do not perpetuate existing inequalities in MH care between ethnic groups.

To address this gap, this study aims to develop and refine an initial programme theory for the important contextual factors and mechanisms that can influence whether acute paediatric settings support ethnic minority CYP in accessing appropriate MH support. This will support the development of equitable MH support in acute paediatric settings and the fulfilment of NHS England’s Core20PLUS5 approach and plan for supporting CYP with MH needs in acute paediatric settings.

The specific question this realist review will answer is ‘How, why and under what circumstances do acute paediatric settings support (or not support) ethnic minority CYP to access appropriate MH support?’.

## Methods and analysis

CYP have varied social, emotional and physical needs, and MH interventions are complex.^30^ In addition, there is diversity within and between ethnic groups.^34^ To account for this complexity, an explanatory realist approach developed by Pawson and Tilly will be used to gain novel insight into how, why and under what circumstances acute paediatric settings support (or do not support) ethnic minority CYP to access appropriate MH support.^35 36^ Unlike traditional reviews, realist reviews are theory-driven and use context-mechanism-outcome (CMO) configurations to explain how interventions influence a mechanism within a particular context to produce an outcome.^36^ In this review, a mechanism is defined as an underlying entity, process or structure that operates in particular contexts to generate outcomes of interest.^37^ Also, ‘context’ refers to pre-existing structures that can influence a mechanism and ‘outcomes’ are anything caused by a mechanism.^38 39^ By focusing on the CMO relationship, realist reviews explain and help predict the varying outcomes of a single intervention.^36^ This insight will guide further exploration through a realist evaluation and inform the development of recommendations that address factors that can affect ethnic minority CYP accessing MH support in clinical practice.

**Figure 1:**
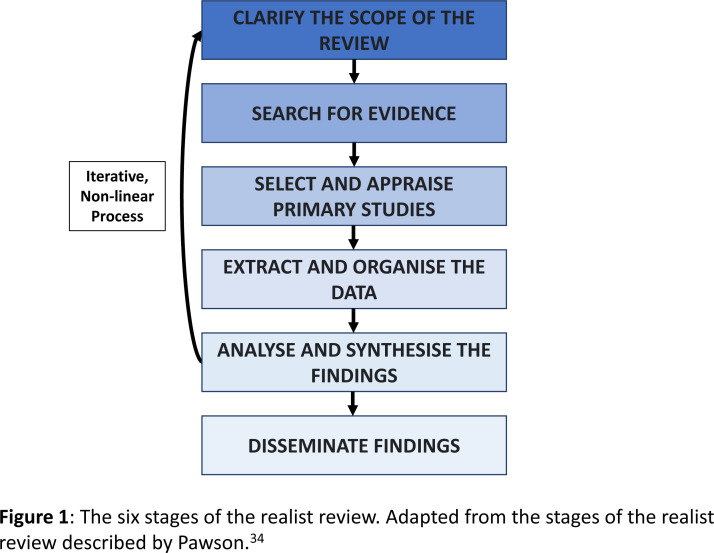
The six stages of the realist review. Adapted from the stages of the realist review described by Pawson.^40^

This realist review will be completed using the six stages described by Pawson, which are outlined in figure 1.^40^ Although the steps are listed in sequence, the iterative nature of realist reviews means they can be revisited and occur concurrently. The RAMESES quality standards for realist synthesis will also be used.^41^ These are widely accepted and recognised quality standards for what constitutes methodological rigour in a realist synthesis.

### Step 1: clarifying the scope of the review

The first step will involve clarifying the scope of the review. This will be achieved through an exploratory search of all the existing literature available in English, with additional sources being identified through snowballing and citation tracking. The findings will then be discussed with the project team and advisory group to refine the review question and purpose and develop a list of initial programme theories. Theories attempt to organise facts within a domain of inquiry into a structurally logical system.^42^ Programme theories are abstracted descriptions and/or diagrams that outline what a programme or intervention is composed of and how it should work. These theories explain how and why outcomes vary between contexts.^43^ The advisory group will consist of professionals involved in CYP’s MH care, ethnic minority CYP with lived experience of accessing MH care in acute paediatric settings and/or their parents/carers. The discussions will involve the groups using their experiential and/or professional knowledge to refine and prioritise factors for the programme theory. The agreed-upon theories will be combined to develop an initial evaluative framework that can be populated with evidence. The theories will be iteratively tested and refined throughout the realist review process. As realist reviews involve an iterative process, Pawson *et al* advise against publishing realist review protocols with a fixed question and purpose.^36^ The refinement process will be ongoing throughout the review, and the scope and theories of the review will be reassessed as new knowledge arises.

To determine the members of the advisory group that would be required, an exploratory search was conducted to identify the ethnic minority groups the review should focus on. The review will focus on specific ethnic groups to gain an appreciation for the variation within the groups. A review of sources of error and bias in ethnicity data collection in the NHS advised that the broad high-level ethnic categories of ‘White’, ‘Mixed’, ‘Asian’, ‘Black’ and ‘Other’ conceal detailed information about the subgroups within the ethnic categories.^44 45^ This is significant as a poor understanding of the nature of ethnic variations in MH is attributed to persisting inequalities in access to MH care for ethnic minorities.^46^ For example, a 2024 systematic review and narrative synthesis concluded that the risk of MH problems for ethnic minority children and adolescents in the UK was comparable to their white counterparts. However, the authors acknowledged that the findings may have been oversimplified by the broad, inconsistent ethnic categories used in the papers included in the review, which may have masked the heterogeneity within the ethnic groups and the unique risks of having MH problems within specific ethnic subgroups.^47^

The search revealed that in England, Black ethnic groups have had persistently higher rates of serious MH issues and detention under the MHA, but poorer access to and outcomes from MH support.^48^ Black ethnic groups were over 3.5 times more likely to be detained under the MHA than their White counterparts and have been found to have disproportionate amounts of force used against them on the wards.^18 49^ They are also less likely to access alternative therapies and psychological interventions.^50^ Additionally, a recent cohort study of all admissions to medical wards in England for CYP aged 5–18 years old from 2012 to 2022 showed CYP from a Black ethnic group made up the lowest proportion of admissions for a MH concern, but had the highest adjusted OR for a MH admission lasting longer than 1 week. The adjusted ORs showed an 81% increased likelihood of a MH admission lasting longer than 1 week for CYP in the Black ethnic group compared with CYP in the White ethnic group.^25^ These findings were discussed with the supervisory team, and it was agreed that a focus on Black ethnic minority groups would be significant for addressing ethnic inequalities in mental healthcare for CYP.

An illustrative and preliminary overarching programme theory statement proposes: ‘*If the acute hospital trust does not support staff to carry out patient-centred psychosocial assessments, have a clear and timely pathway for referring CYP for MH support, or offer a diverse range of MH support options (Context), then Black and Mixed-Black CYP will be less likely to access appropriate mental health support (Outcome), because staff will find it challenging to carry out patient-centred psychosocial assessments and refer CYP for the appropriate support (Mechanism*).’ This statement relates to the capacity of acute hospital trusts to support CYP from Black and Mixed-Black ethnic groups to access culturally appropriate MH support in acute paediatric settings. This preliminary statement has been formed through an exploratory literature review, consultation with the advisory group and public involvement at community events. Relevant granular contextual factors that have contributed to this illustrative statement include the availability of appropriate translation services and cultural competency training for staff, and Trust partnerships with relevant individuals and organisations from Black and Mixed-Black communities.^51^

Ethnicity-related factors will primarily be viewed as contextual factors, and the review will endeavour to theorise how their interaction with structural factors at a micro-level, meso-level and macro-level may trigger mechanisms to produce outcomes relevant to access to MH support in acute paediatric settings.^52^ To prevent essentialism, the review will avoid theorising the relationship between ethnicity and outcomes related to access in isolation from structural factors.^53^ Within the programme theory, the review will aim to distinguish ethnicity-related factors from pre-existing structural factors by comparing and contrasting relevant findings for Black and Mixed-Black groups with those from majority groups. The interaction between ethnicity and wider structural factors is complex, and the availability of evidence and the nature of study reporting will be a limiting factor in this process. However, it will be supported through counterfactual reasoning in discussions among the research team and advisory group.^54^ This will involve the consideration of whether a mechanism would still be triggered if the ethnicity of the CYP and/or their parent, or the wider structural context were changed. Additionally, the review will inform a primary study, which can use primary data to explore aspects of the programme theory that are not available in the literature. If the evidence allows, the review will also consider intersectional differences within Black and Mixed-Black ethnic groups by reporting the results of the heterogeneous groups within the broader categories separately, rather than solely combining them.

### Step 2: searching for evidence

The next step will involve a more thorough literature search to identify evidence to test and refine the initial programme theories and identify potential new relevant theories. It will also provide a deeper insight into the literature on the topic. The search terms will be determined by the research team and based on the list of theories selected in Step 1.

The electronic databases we will search, but not be limited to, are OVID Medline, PsycINFO, CINAHL and SCOPUS. Relevant data will also be sought through snowballing, backward citation searching on included studies, document recommendations from professionals involved in CYP’s MH, and the research team. Realist reviews include a wider range of evidence than traditional reviews, so grey literature and policy review searches on Grey Matters, Health Management Information Centre and Google Scholar will be conducted to gain a deeper insight into the relevant contexts and mechanisms of the interventions. Documents featuring Black and/or Mixed Black CYP with MH issues in acute paediatric settings will be included. Table 1 outlines the potential inclusion and exclusion criteria. There will be an initial focus on outcomes that relate to the disclosure, assessment and management of MH issues in Black and Mixed/Black CYP. The outcomes will be iteratively refined and prioritised by consulting the literature and advisory group.

**Table 1:** Potential inclusion and exclusion criteria

Inclusion criteria	
Population	CYP (<18 years old) from Black and/or Mixed Black ethnic groups with MH issues
Setting	Acute paediatric settings; Paediatric ward, Emergency Department Evidence available in English will be included for review. The reporting will focus on the geographical context of England
Exclusion criteria	
	Papers unavailable in English Papers that do not include Black ethnic groups Papers that do not include CYP (<18 years old) Papers that include CYP with other age groups and do not report the results separately Papers that do not report the findings of ethnic groups separately

CYP, children and young people; MH, mental health.

The review will focus on English-only literature despite the potential for bias, because the review will inform a primary study that will be based in England. There will not be a limit on the date of publication.

Unlike traditional reviews, the search strategy in realist reviews is iterative and will evolve as the research team’s understanding of the topic grows.^36^ Multiple searches will be conducted throughout the review, with a final search near the end of the study to identify any new relevant data. Our initial literature search revealed limited literature on CYP from Black and Mixed-Black ethnic groups accessing MH support in acute paediatric settings. It is anticipated that the search will be broadened to include primary, community and hospital MH settings and/or access to support for other conditions in acute paediatric settings and/or the MH support available to all CYP in acute paediatric settings to develop the programme theory. An example search strategy is provided in the online supplemental material.

The goal of reviewing literature for realist reviews is to reach a point of theoretical saturation.^36^ This is thought to be reached when the literature retrieved from a search cycle fails to add additional insight into the intervention, and further searches are unlikely to do so either.^36^ Search results will be stored in Rayyan for sorting, and a copy of the search results will be stored on a secure server.^55^

### Step 3: selection and appraisal of primary studies

All the papers will be screened by one researcher in Rayyan, and 10% of the papers will also be screened by a second researcher for calibration and quality control purposes.

The title and abstract of the papers will initially be screened using the inclusion and exclusion criteria in table 1. Included abstracts will subsequently undergo a full-text screen and will be concurrently appraised for relevance. Documents will be deemed relevant if they help build and/or test the theories. Documents that contribute data to multiple CMOs will also be appraised for rigour. Quality appraisal in realist reviews involves assessing the data within the documents that contribute to the programme theory. Rigour is determined by considering if the conclusions drawn by the author have enough methodological credibility to be used to test the theories.^36^ This process may be supported by methodological appraisal checklists. However, there are no strict criteria for quality appraisal in realist reviews, and the relative contribution each source makes to the programme theory will be considered when reviewing evidence for inclusion.^56^ Any disagreements will be resolved through discussion between the two researchers and/or the wider project team.

### Step 4: extraction and organisation of the data

The full texts of the papers and other evidence will subsequently be uploaded into NVivo for organisation and coding. Relevant information within the evidence that contributes to the programme theory development will be highlighted, noted and coded.^36^ Codes will also be made for data that complements or refutes the programme theories so they can be considered when developing the potential pathways for the intervention theories. Comparable to the data selection process, the documents will be coded by one researcher, and a random 10% subset of the documents will also be coded by a second researcher for consistency. Any disagreements in the coding process will be resolved through discussion between the two researchers and/or the wider project team. Descriptive data for the included documents will be recorded in Microsoft Excel. This will consist of the first author, year, document type, aim, study design, population, intervention, outcomes measured and the section of the programme theory the document contributes to.

Like the evidence search, this is an iterative process. The evidence will be reviewed, and data will be extracted multiple times throughout the review.^36 57^ A record of the methods used to omit or use evidence and how and why the programme theories have changed will be kept in Microsoft Excel and NVivo.

### Step 5: analysis and synthesis of findings

Once extracted, the data will be synthesised to refine the programme theory into a middle-range theory. A theory is deemed ‘middle-range’ when it is abstracted at the level of CMO configurations, making it testable. The extracted data will be categorised into key themes, and the data underpinning the key themes will be organised into CMO configurations. CMO configurations aim to reveal the fundamental generative causal mechanisms that explain how and why outcomes arise in particular contexts.^58^

Retroduction will be used to develop these CMO configurations and theorise programmes. Retroduction involves inductive reasoning, which develops new theories based on observations, and deductive reasoning, which tests theories with evidence to identify the hidden underlying causal mechanisms that generate outcomes in particular contexts.^59^ This process will occur at two levels of analysis. The first level will determine the contexts within which the mechanisms are and are not triggered, and the second level will explain the pattern of the contexts, mechanisms and outcomes.^43^ Discussions of the experiential and professional experiences of the review team and advisory group will enable the research team to comprehend the data from a broad perspective and distinguish contextual factors from mechanisms in the analytic process. This will involve the consideration of whether the identified factors relate to pre-existing components of the setting or system that Black and Mixed-Black children access MH support within (context), or an underlying reasoning and response that triggers the relevant outcome (mechanism).^37 38^

Consistency in the analysis and synthesis of the findings will be managed by using the same definitions for ‘context’, ‘mechanism’ and ‘outcome’ throughout the review, anchoring data analysis to the initial programme theory, and keeping a record of theory refinement throughout the review.

To adjudicate between any competing theories, the consilience, simplicity and analogy of the programme theories will be considered. Consilience is when a theory explains a wider breadth of evidence than the competing theories, simplicity refers to a theory being easy to understand without requiring additional assumptions to explain the data. Also, analogy relates to the theory being consistent with what is already known, such as an existing substantive theory.^60^

The involvement of the advisory group and wider research team who have lived and professional experience of the phenomenon, and inclusion of grey literature in the analytic process, will support analytic coherence being maintained if a small evidence base is identified from the white literature. Furthermore, this review is a precursor to a primary study, where elements of the programme theory that are not adequately reflected in the literature can be explored further using primary data.

### Step 6: dissemination

The middle-range theory will inform a primary realist evaluation study, where the theory will be tested and refined further. A lay summary of the review findings will be shared directly with the advisory group, and they will be encouraged to share the findings with their networks. The findings will also be disseminated through publication in an open-access journal and presentations at relevant research conferences. Any important amendments to the protocol will be reported in PROSPERO and/or the final review manuscript.

### Patient and public involvement

The study’s conceptualisation was inspired by the authors’ interactions with CYP with lived experience of MH issues and their families during their professional practice. An advisory group of key stakeholders, including ethnic minority CYP with lived experience of MH issues, their families and professionals involved in their care, will be consulted throughout each stage of the review. This will support the middle-range theory to reflect the experiences of ethnic minority CYP with MH issues and their families, the professionals involved in their care and acute paediatric settings.

So far, professionals involved in CYP’s MH care, a child from a Mixed-Black ethnic group with lived experience of accessing MH support in acute paediatric settings and their parent have been recruited to the project’s advisory group. They have supported decision-making related to the project’s scope, search strategy and initial programme theory. Recruitment efforts to date have involved members of the research team approaching their existing networks, pre-existing patient and public involvement groups and relevant charities. Public input on the review has also been sought through delivering talks, a workshop targeted at CYP, and networking at community events targeted at Black ethnic groups. MD-S-E has facilitated discussions and reached a consensus in the review decision by encouraging participation from all individuals and introducing viewpoints that have been raised in the literature and by other advisory group members in an unbiased manner. If any differences in opinion were to occur that required further reconciliation, input and guidance would be sought from the wider research team.

The recruitment of advisory group members will be an ongoing and agile process that is regularly reviewed with existing advisory group members to encourage representation across Black and Mixed-Black ethnic groups.

## Ethics and dissemination

This realist review will only involve secondary data, so ethical approval will not be required.

The output of the review will be a middle-range programme theory that will inform a primary study, which will test and refine the theory further. The review findings will be disseminated through publication in a peer-reviewed journal and presentations at research conferences and stakeholder events. A lay summary of the findings will also be shared with the advisory group members, who will be encouraged to share the findings with their networks.

## Supplementary material

10.1136/bmjopen-2025-104145 online supplemental file 1
