# Supplementary material for: Exploring and Modifying BarrieRs to enhance ACcess to mental health support for Ethnic minority Children and Young People (CYP) in acute paediatric settings (EMBRACE) in England: a realist review protocol
Source: BMJ Open. 2026 Jan 22;16(1):e104145. doi: 10.1136/bmjopen-2025-104145 (PMC12829346; doi:10.1136/bmjopen-2025-104145)
Supplement: online supplemental file 1 [file bmjopen-16-1-s001.docx]

**EMBRACE OVID MEDLINE Search Strategy:**

|  | Search terms: |
| --- | --- |
| Black/Mixed Black | ("BAME" or BME or "Black Asian" or "minority ethnic*" or "ethnic minorit*" or "people of color" or "people of colour" or POC or "racial* minorit*" or "Race Factor*" or "Mixed rac*" or "Mixed Black" or minorit* or "ethnic* group*" or "Black British" or "African people" or "afro caribbean" or "Caribbean people" or "african caribbean" or afrocaribbean or "afro-caribbean" or "Black Caribbean" or "Black African" or "multiracial person" or migrant or "Black People" or "Black or African American" or "race difference" or "ethnic difference" or "racial groups" or "Cultural Characteristics" or "Cultural Diversity" or "Cross-Cultural Comparison").ti,ab,kf.  OR  ((racial adj5 disparit*) or (ethnic adj5 disparit*)).ti,ab,kf.  OR  ((minority adj1 (group* or population*)) or multicultur* or multi cultur* or multiethnic* or multi ethnic* or ((cultur* or ethnic* or racial*) adj1 (divers* or differen*))).ti,ab,kf. |
| CYP | (teen* or youth* or adolescen* or juvenile* or child* or “young adult”* or “young people” or “young person” or highschool* or college* or girl* or boy* or paediatric patient* or pediatric patient*).ti,ab,kf. |
| Mental health issues | (depression* or self-harm or suicidal or "depressive disorder*" or eating disorder* or suicide or "mental needs" or mental distress or bipolar or "elimination disorders" or schizophrenia or psychotic or "substance-related disorders" or "trauma and stressor related disorders" or "psychiatric case" or "psychiatric patient" or CAMHS or anxi* or depress* or OCD or "obsessive compulsive" or "attention deficit" or ADHD).ti,ab,kf.  OR  ((mental or mentally or behavioural or emotional or psychology* or psychiatr* or behaviour or emotion* or affective or mood or eat* or conduct or personality or somatic or somatoform) adj3 (health or disease or disorder* or ill* or illnesses or abnormality or abnormalities or disturbance or disturbances or problem or problems or condition or conditions or diagnos* or symptom* or "well-being" or wellbeing or syndrome*)).ti,ab,kf. |
| Acute paediatric setting | (a&e or emergency department or children* unit or children* ward* or (("non psychiatric" or pediatric or paediatric or "acute physical") adj2 (department* or "health facility*" or hospital* or unit* or ward* or admission or setting or inpatient*))).ti,ab,kf.  OR  (accident adj1 emergency).ti,ab,kf. |
