# Supplementary material for: Exploring and Modifying BarrieRs to enhance ACcess to mental health support for Ethnic minority Children and Young People (CYP) in acute paediatric settings (EMBRACE) in England: a realist review protocol
Source: BMJ Open. 2026 Jan 22;16(1):e104145. doi: 10.1136/bmjopen-2025-104145 (PMC12829346; doi:10.1136/bmjopen-2025-104145)
Supplement: Supplementary file 2 [file bmjopen-2025-104145.reviewer_comments.pdf]

PEER REVIEW HISTORY

BMJ Open publishes all reviews undertaken for accepted manuscripts. Reviewers are asked to complete a checklist review form and are provided with free text boxes to elaborate on their assessment. These free text comments are reproduced below.

ARTICLE DETAILS

Title (Provisional)

Exploring and Modifying BarrieRs to enhance ACcess to mental health support for Ethnic minority Children and Young People (CYP) in acute paediatric settings (EMBRACE) in England: a realist review protocol

Authors

Da-Silva-Ellimah, Morenike; Darko, Natalie; Kotera, Yasuhiro; Sayal, Kapil; Manning, Joseph C

VERSION 1 - REVIEW

**Reviewer**	**1**
**Name**	**Awara, Mahmoud**
**Affiliation**	**Dalhousie University, Psychaitry**
**Date**	**06-Dec-2025**
**COI**	**None**

Thank you for inviting me to review this interesting protocol.

Overall, this protocol addresses a critically important area but requires additional conceptual and methodological clarity before publication.

-The protocol does not explain how ethnic disparities will be examined when mental health services are universally insufficient for all CYP.

-It is unclear how ethnicity-specific mechanisms will be distinguished from general system-wide pressures.

-The manuscript does not clarify how contextual factors (e.g., long waits) will be separated from mechanisms (e.g., mistrust or perceived discrimination) in the realist analysis.

-No illustrative CMO examples are provided, limiting insight into how the authors intend to construct or test explanatory pathways.

-The protocol does not address how heterogeneity within Black and Mixed-Black groups (e.g., African vs Caribbean backgrounds) will be considered.

-Intersectional factors such as socioeconomic status, migration history, and disability are acknowledged but not incorporated into the analytic plan.

-Restricting the review to English-language publications may introduce bias and exclude relevant evidence.

-The inclusion criteria may yield few eligible studies, yet the authors do not outline how they will maintain analytic coherence with a small evidence base.

-Coding and synthesis procedures are described briefly, without detail on how consistency, disagreements, or limited evidence will be managed.

-The advisory group section does not explain how diverse representation across Black and Mixed-Black subgroups will be ensured or how differing views will be reconciled.

-The Delphi process is referenced but not operationalized, leaving uncertainty about consensus procedures.

-The protocol does not clarify how the final theory will distinguish inequities driven by ethnicity from those driven by general structural shortages.

Thank you again for the opportunity to review this realist review protocol.

VERSION 1 - AUTHOR RESPONSE

<u>Reviewer 1 comments to the Author</u>:

Comment: The protocol does not explain how ethnic disparities will be examined when mental health services are universally insufficient for all CYP.
Response: An explanation has now added to the methodology section. Dependant on the availability of evidence and nature of study reporting, the review will aim to examine ethnic disparities by comparing and contrasting relevant findings for Black and Mixed-Black groups with those from majority groups. It is recognised that this process will be dependent on the availability of sufficient evidence and study reporting, but this process will be counterfactual reasoning in discussions amongst the research team and advisory group. Also, the study will inform a primary evaluation, which can explore aspects of the programme theory that are not available in the literature.
Revision location: Step 1: Clarifying the scope of the review

Comment: It is unclear how ethnicity-specific mechanisms will be distinguished from general system-wide pressures.
Response: This has now been explained in the methodology section. Ethnicity related factors will primarily be viewed as contextual factors, and the review will endeavour to theorise how their interaction with structural factors such as system pressures may trigger mechanisms to produce outcomes relevant to access, rather than the effect of ethnicity on access alone, to avoid essentialism. Within the programme theory, ethnicity related factors will be distinguished from structural factors such as healthcare system pressures by comparing and contrasting relevant findings for Black and Mixed-Black groups with those from majority groups. It is recognised that this process will be dependent on the availability of sufficient evidence and study reporting, but this process will be counterfactual reasoning in discussions amongst the research team and advisory group. Also, the study will inform a primary evaluation, which can explore aspects of the programme theory that are not available in the literature.

Revision location: Step 1: Clarifying the scope of the review

Comment: The manuscript does not clarify how contextual factors (e.g., long waits) will be separated from mechanisms (e.g., mistrust or perceived discrimination) in the realist analysis.
Response: Thank you for your comment. Further detail on how this will be achieved through considering if the factors relate to the pre-existing components of the setting or system (context), or an underlying reasoning and response that is triggered to produce the relevant outcome (mechanism) has been added. This process will be supported by consultation with the advisory group.
Revision location: Step 5: Analysis and synthesis of findings

Comment: No illustrative CMO examples are provided, limiting insight into how the authors intend to construct or test explanatory pathways.
Response: A preliminary and illustrative overarching programme theory statement has been added to the 'Methods and analysis' section to provide further insight.
Revision location: Step 1: Clarifying the scope of the review.

Comment: The protocol does not address how heterogeneity within Black and Mixed-Black groups (e.g., African vs Caribbean backgrounds) will be considered.
Response: This has now been explained in the methodology section. If the evidence allows, the review will address intersectional differences within Black and Mixed-Black ethnic groups by separately reporting the results of heterogenous groups. Also, the search strategy has been broadened to include documents that feature Black and Mixed-Black CYP with mental health issues in an acute paediatric setting to support this.
Revision location: Step 1: Clarifying the scope of the review, Step 2: Searching for evidence.

Comment: Intersectional factors such as socioeconomic status, migration history, and disability are acknowledged but not incorporated into the analytic plan.
Response: This has now been explained in the methodology section. If the evidence allows, the review will address intersectional differences within Black and Mixed-Black ethnic groups by separately reporting the results of heterogenous groups. Also, the search strategy has been broadened to include documents that feature Black and Mixed-Black CYP with mental health issues in an acute paediatric setting to support this.
Revision location: Step 1: Clarifying the scope of the review, Step 2: Searching for evidence.

Comment: Restricting the review to English-language publications may introduce bias and exclude relevant evidence.
Response: Thank you for your comment. Whilst the potential for bias is recognised, the review focuses on English-Only literature as the review is being conducted to inform a primary evaluation that will be based in England. An explanation of this has been added to the manuscript.
Revision location: Step 2: Searching for evidence.

Comment: The inclusion criteria may yield few eligible studies, yet the authors do not outline how they will maintain analytic coherence with a small evidence base.
Response: A description has been added to the 'Analysis and synthesis of findings' section. Analytic coherence will be supported by the inclusion of grey literature and consultation with the wider research team and advisory group. Also, the study will inform a primary evaluation, which can explore aspects of the programme theory that are not available in the literature. After discussion with the wider supervisory team and an independent panel at the host institution, analytic

coherence has also been supported by rephrasing the study aims to better reflect the realist methodology and the phenomena that is being theorised.
Revision location: Step 5: Analysis and synthesis of findings.

Comment: Coding and synthesis procedures are described briefly, without detail on how consistency, disagreements, or limited evidence will be managed.
Response: Thank you for your comment. Further detail on how consistency, disagreements, or limited evidence will be managed have been added to the 'Extraction and organisation of the data' 'Analysis and synthesis of findings' section.
Revision location: Step 4: Extraction and organisation of the data, Step 5: Analysis and synthesis of findings.

Comment: The advisory group section does not explain how diverse representation across Black and Mixed-Black subgroups will be ensured or how differing views will be reconciled.
Response: A diverse representation across Black and Mixed-Black subgroups in the advisory group will be encouraged through an ongoing and agile recruitment process. The 'Patient and Public Involvement' section has been revised to include further detail on this process and how differing views will be reconciled.
Revision location: Patient and Public Involvement.

Comment: The Delphi process is referenced but not operationalized, leaving uncertainty about consensus procedures.
Response: The Delphi process has been removed from 'Step 1: Clarifying the scope of the review', as it was not perceived as adding sufficient value in light of the iterative nature of the realist review.
Revision location: Step 1: Clarifying the scope of the review

Comment: The protocol does not clarify how the final theory will distinguish inequities driven by ethnicity from those driven by general structural shortages.
Response: This has now been explained in the methodology section. Ethnicity related factors will primarily be viewed as contextual factors, and the review will endeavour to theorise how their interaction with structural factors such as system pressures may trigger mechanisms to produce outcomes relevant to access, rather than the effect of ethnicity on access alone, to avoid essentialism. Within the programme theory, ethnicity related factors will be distinguished from structural factors such as healthcare system pressures by comparing and contrasting relevant findings for Black and Mixed-Black groups with those from majority groups. It is recognised that this process will be dependent on the availability of sufficient evidence and study reporting, but this process will be counterfactual reasoning in discussions amongst the research team and advisory group. Also, the study will inform a primary evaluation, which can explore aspects of the programme theory that are not available in the literature.
Revision location: Step 1: Clarifying the scope of the review.
